# Comparison of Electronic Fruits for Impact Detection on a Laboratory Scale

**DOI:** 10.3390/s130607140

**Published:** 2013-05-30

**Authors:** Ulrike Praeger, Jelena Surdilovic, Ingo Truppel, Bernd Herold, Martin Geyer

**Affiliations:** Department of Horticultural Engineering, Leibniz Institute for Agricultural Engineering Potsdam-Bornim (ATB), Max-Eyth-Allee 100, Potsdam 14469, Germany; E-Mails: jsurdilovic@atb-potsdam.de (J.S.); itruppel@atb-potsdam.de (I.T.); bherold@atb-potsdam.de (B.H.); mgeyer@atb-potsdam.de (M.G.)

**Keywords:** electronic fruit, mechanical load, impact acceleration, impact force, drop simulator, processing line simulator, potato

## Abstract

Mechanical loads cause severe damage to perishable agricultural products. In order to quantify the mechanical impact during harvest and postharvest processes, several electronic fruits have been developed. The objective of the work described here was to compare on a laboratory scale different types of impact acceleration recording electronic fruits: Mikras implanted in a real potato tuber as well as in a dummy tuber, IRD, Smart Spud and TuberLog. The acquisition of mechanical impacts was performed using a drop simulator with optional steel or PVC as impact material as well as a processing line simulator. Our results show that drops from 10 cm height on PVC caused similar peak accelerations of Mikras implanted in a real potato or a dummy, IRD and TuberLog. When dropped onto steel however, IRD, TuberLog and Mikras implanted in a dummy recorded higher peak values than Mikras in real potatoes. Impact on the flat side of a tuber led to higher peak values than impact on the apical region. This could be caused by different elastic compliance of synthetic materials as well as material thickness. Running through the processing line simulator TuberLog recorded the most impact; Smart Spud recorded a low number of impacts compared to the other electronic fruits. In all experiments the least sensitive measurements were recorded using Smart Spud.

## 1. Introduction

Mechanical impact during harvest and postharvest processes causes external as well as internal damage to agricultural and horticultural products, e.g., black spot disease of potatoes. This damage decreases the quality of the product. Increased mechanization and accelerated flow rates of the produces during the harvest and postharvest processes increase the mechanical impact and thus increase product losses.

In the last 40 years ‘artificial’ or ‘electronic fruits’ equipped with miniaturized electronic sensors have been developed to measure impacts during harvest and postharvest processing lines such as transport, grading and packaging of perishables. Their goal is to detect critical impact points on the product, as well as to optimize processing lines in order to reduce product damage.

Since the design of the first artificial ‘pseudo-fruit’ in California [1] numerous devices have been developed. Typically their shape tries to imitate that of the real product, with some being shaped as spheres to imitate fruits such as apples, lemons, peaches. Others have longish shapes imitating tubers such as potatoes. Some impact measurement techniques can use differently shaped artificial fruits (IRD, Techmark Inc., Lansing, MI, USA) or Smart Spud (Sensorwireless, Charlottetown, PEI, Canada). Electronic fruits with different sensory principles exist; some being able to measure acceleration, as well as pressure. Data acquisition of these devices is radio-transmitted to a base station, or stored internally in the device itself using a data logger. The instrumented sphere IS100 was the first acceleration recording device for acquisition of mechanical impacts on apple fruit equipped with microprocessor technology developed by researchers of Michigan State University and manufactured by Techmark Inc. [2]. The pressure measuring sphere PMS-60 (Magnettech, Berlin, Germany) has a spherical form and was used amongst others for determining damage of apples and onions during postharvest handling [3,4]. BIRD (Berry Impact Recording Device) is a miniature instrumented sphere for measuring impact acceleration of small fruits such as blueberries. The BIRD sensor is cast in silicone rubber as an ‘instrumented sphere’ with 25.4 mm in diameter, developed by a research team in Georgia (USA) [5].

The great economic importance of reducing damage, e.g., black spot disease, of potatoes, has driven creation of several longish-shaped acceleration measuring devices trying to mimic potato tubers. These include the IRD (Techmark), PTR-200 (SM Engineering, Nakskov, Denmark) [6], Smart Spud (Sensor Wireless) [7] and recently TuberLog (ESYS GmbH, Berlin, Germany).

Damage susceptibility of perishables after mechanical load varies and it depends on many factors such as variety, cultural practices, storage duration and temperature among others. Numerous laboratory tests have been performed in order to study the relationship between on the one side peak acceleration and velocity change measured by electronic fruits and on the other side the mechanical damage inflicted to the biological produce. In this way, thresholds of allowable mechanical impact loads have been determined [8–10]. These threshold values are necessary in order to interpret the recorded acceleration values during a postharvest process for prediction of the produce bruise probability. Studies have also been done to link the measurements of impact devices in processing lines of agricultural products to the specific damage caused by these impacts [11–13].

For determination of realistic mechanical loads inflicted on the produce, measuring devices which are similar to the real products concerning the geometrical shape and the physical properties like the elastic compliance are necessary. They should be able to imitate the path of motion of the real product in order to be able to record a realistic number of impacts that happen throughout different phases in a processing line. A miniaturized acceleration measuring unit Mikras has been developed (ESYS GmbH), which can be implanted in different real products such as fruits or tubers. Mechanical impacts are then measured under consideration of the shape and the physical properties of the real plant tissue [14].

The objective of this work was to compare the impact acceleration data recorded with several electronic fruits and the Mikras device in the real product. Impacts of the systems IRD, Smart Spud with two different shapes, TuberLog and Mikras, implanted in a synthetic dummy tuber and a real potato tuber have been measured. Free-fall experiments for measuring the impact acceleration from defined heights onto steel and PVC foam as well as impact measurements using a laboratory processing line simulator have been carried out. For data evaluation of the free-fall experiments the peak acceleration of the impacts was used. For the analysis of the processing line simulation the frequency distribution of impacts according to the peak acceleration, the number of recorded impacts and the duration of the runs were determined.

## 2. Experimental Section

### 2.1. Measuring Devices

In this section we will describe four impact measurement devices that were used during laboratory tests. Table 1 presents the technical data gathered for each of those devices as well as specific settings applied during performed experiments. All measuring devices were used with the calibration provided by the respective manufacturers.

#### 2.1.1. Mikras

**Figure 1.**
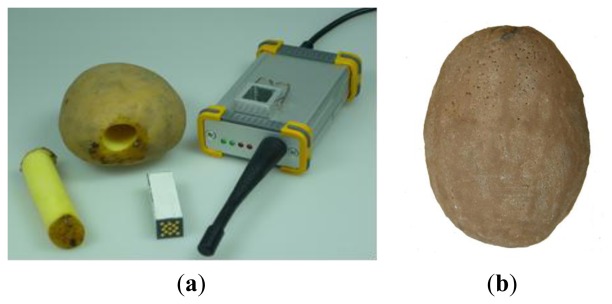
(**a**) Mikras acceleration measuring unit before insertion in a real potato with adaptor for data readout (**b**) Synthetic potato dummy from Grimme company in which Mikras was implanted.

Mikras (ESYS GmbH) is a miniaturized triaxial acceleration measuring unit that is implanted in real agricultural product of adequate size (Figure 1). For these tests a refined measuring unit with internal data storage was used instead of wireless data transmission of the previous device [14]. Dimensions and mass of the Mikras are 42 × 13 ×13 mm and 14 g (density of 2 g·cm^−3^), respectively. The device contains a rechargeable NiMH battery, a data acquisition system and the triaxial acceleration sensor. The components are encased in epoxy resin and a ceramic coating. After the measurements stored data (acceleration courses and peak values of each impact event) are transferred by plugging the measuring unit into a PC-USB-adaptor.

In our tests we implanted Mikras in real potato tubers as well as in a potato tuber dummy made of polyurethane (Figure 1), which was provided to us by Grimme Landmaschinenfabrik (Damme, Germany). The density of the synthetic dummy was 1.14 g·cm^−3^ which is similar on average to real potato tubers (1.08 g·cm^−3^). The potato tubers of cultivar ‘Karlena’ with a round shape used were harvested in September 2009 in the Mecklenburg-Vorpommern region in Germany from a commercial grower and stored 8 months at 4 °C. The mass of the potato tubers increased about 3% after implanting the device.

#### 2.1.2. IRD

**Figure 2.**
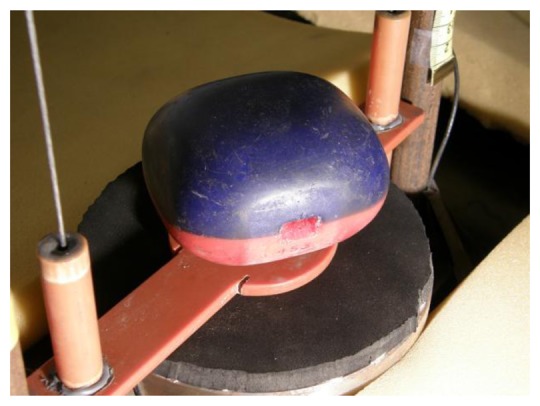
IRD on the sliding carriage of the drop simulator (Section 2.2.1).

Impact Recording Devices (IRD, Techmark) are the most commonly used electronic devices for acquiring mechanical load measurements of agricultural products. IRD have been utilized for over 20 years with some most notable versions being the IS 100 (instrumental sphere [2,15]) and later IRD-400 which is available in multiple shapes such as sphere and flattened sphere (Figure 2).

The IRD-400 consists of a tri-axial accelerometer sensor, a microprocessor for data collection, an internal real-time clock, and a rechargeable Ni-Cd battery [16]. The electronic part is placed in a comparatively hard synthetic casing.

When using IRD devices, peak acceleration and velocity changes during single impact are measured and used to estimate the bruise probability of the tested products. The calculated damage boundary values are then used to indicate whether impacts caused damage to the products during their run through the processing line. The complete acceleration course values during test measurements are also obtainable and can be used for data interpretation.

#### 2.1.3. Smart Spud

**Figure 3.**
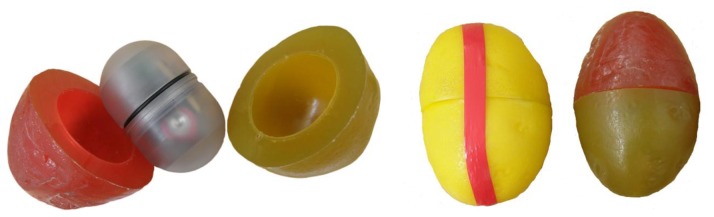
Smart Spud sensor with original casing from the Sensor Wireless company (red-green) and casing from the Grimme company (yellow).

The Smart Spud measuring system was manufactured in 2000 by Sensor Wireless. For the measurements of impact acceleration different urethane casings can be chosen, into which the sensor is embedded. In our tests a shape originally manufactured by Sensor Wireless and another more firm polyurethane shape, fabricated by Grimme Landmaschinenfabrik, were used (Figure 3). Smart Spud is powered by a user-replaceable lithium battery. Data measurements can be monitored on a hand-held wireless computer. A monitor on a palm handheld shows peak acceleration of each impact event continuously during the measurement [17].

#### 2.1.4. TuberLog

**Figure 4.**
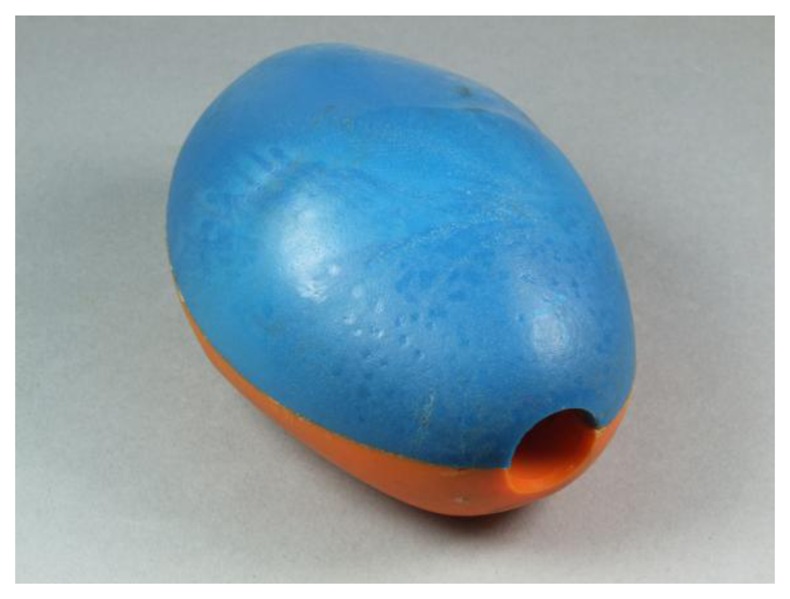
TuberLog acceleration measuring device.

The TuberLog system was developed in 2011 by ESYS GmbH. It is used to measure the mechanical load of potato tubers during their harvesting and postharvest processes. A triaxial impact acceleration sensor is embedded in a synthetic potato shaped device (Figure 4) with length of 90 mm. The data logger inside the TuberLog is supplied by a rechargeable lithium-ion battery. The communication for starting measurements and data readout of the data logger can be accomplished over a bluetooth interface or via USB. Data measured by the Tuberlog device contains peak acceleration values over the measured time of the harvesting processes.

### 2.2. Test Facilities

#### 2.2.1. Drop Simulator

A drop simulator which is described in detail in Geyer *et al.* [14] was used to measure impact acceleration when dropping the electronic fruits or potato tubers onto a steel plate from variable heights up to 1 m. The electronic fruit or tuber is dropped by manual placing it into a circular hole (diameter 40 mm) in the middle of a free-falling sliding carriage and pre-setting its drop direction.

Underneath the steel plate a sensor is placed which measures the course of the impact force with a scanning rate of 10 kHz. With the measured data we can then calculate the peak force and the duration and integral of the impact. When testing with different impact materials the steel plate can be covered with suitable cushioning pads, e.g., PVC foam.

#### 2.2.2. Processing Line Simulator

**Figure 5.**
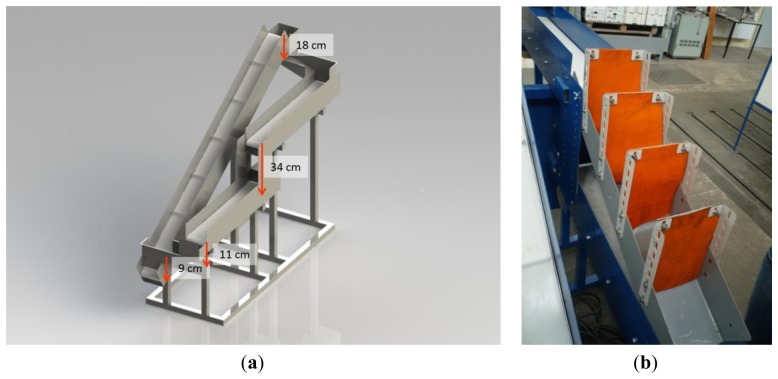
(**a**) Processing line simulator for conveying agricultural products after harvest. (**b**) Inserted chute at the highest drop step.

A continuously moving laboratory processing line was used for simulating mechanical load in a postharvest chain. Products pass through a line with three conveyor belts (one drag conveyor belt with ascending slope of 30.5° and two conveyor belts in horizontal position), four drop steps and two small chutes, with a total conveyor length of 5.2 m. The drop steps have a total height of 72 cm, the highest one being 34 cm (Figure 5). The processing line could be modified at the highest falling step. To accomplish this, a chute with four retaining blankets was inserted in order to slow down the tuber's velocity (Figure 5).

### 2.3. Test Conditions

#### 2.3.1. Drop Simulator

Ten drops of the electronic fruits have been performed per test condition. For each, impact acceleration was recorded by the device and the impact force was recorded by the sensor in the drop simulator.

Six different types of specimen were used during testing (Table 1). These included Mikras implanted in a real tuber (cultivar ‘Karlena’) as well as in a potato dummy, IRD, two different shapes of Smart Spud, as well as TuberLog.

Measuring devices were dropped from different heights (10 cm, 25 cm) onto a steel plate and 5 mm thick PVC foam. The PVC foam characteristics are described by the force-displacement diagram in Geyer *et al.* [14].

Due to the upper limit of the force sensor of 600 N drops from 25 cm heights were done only onto PVC foam. The SmartSpud sensor with the original shape was not able to record acceleration values when dropped from 10 cm onto PVC foam so in those cases it was not possible to record the peak force values.

Two drop directions were used for each device, namely longitudinal, whereby the impact happens on the device's apical area, as well as lateral, where the impact happens on their flat side.

#### 2.3.2. Processing Line Simulator

The same six electronic fruits used in drops simulator tests were also used here, each passing the processing line simulator ten times in order to compare the measuring behavior and the type of motion of the electronic fruits. Every run of potato tubers as well as electronic fruits through the processing line simulator can be considered as widely stochastic process. Therefore ten times runs have been used for statistical evaluation. Two different conveyor velocities of 0.19 m·s^−1^ (slow) and 0.35 m·s^−1^ (fast) were used according to practical conditions. Test runs were performed without a chute from 34 cm heights as well with a chute as described in Section 2.2.2.

### 2.4. Data Evaluation

#### 2.4.1. Drop Simulator

Average values of peak acceleration and the peak force of test drops were used to compare mechanical impacts of the tested electronic fruits. Calculated standard deviation values are depicted as error bars in the produced diagrams.

The damage dealt to a product at its impact positions is related to the impact force depending on its mass rather than the impact acceleration measured by the electronic fruits. In order to compare the electronic fruits concerning their impact force estimation we calculated relationships between the peak force measured by the drop simulator and the peak force calculated via peak acceleration data measured by electronic fruits using the following function:

(1) $$\frac{F_{m}}{{\hat{F}}_{c}}$$

where:

*F_m_* = peak force measured by the drop simulator
*F̂*_*c*_= m * *â* = calculated equivalent peak force
*m* = mass of electronic fruit or potato tuber (with implanted Mikras)
*â* = peak acceleration measured by electronic fruit

#### 2.4.2. Processing Line Simulator

Both the amount of time and number of impacts with peak accelerations greater or equal to 30 g were calculated for each of the test runs. Frequency distribution of impacts according to the peak acceleration measured was calculated using a triangular kernel density estimator.

## 3. Results and Discussion

### 3.1. Impact Measurement of Falls with Defined Drop Height

**Figure 6.**
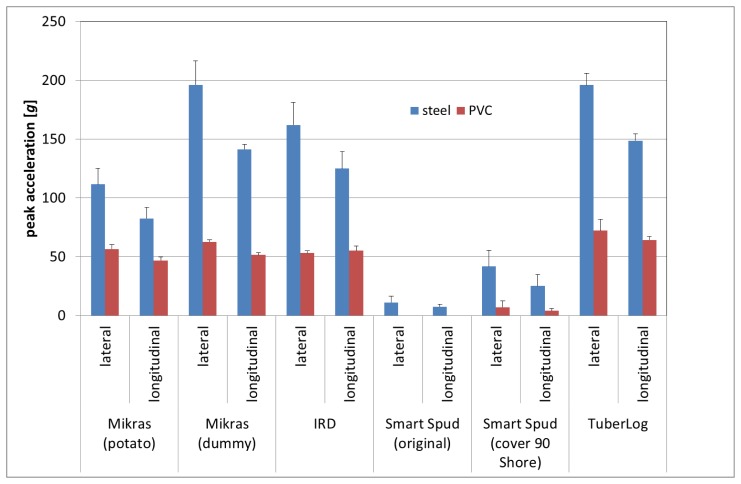
Peak acceleration measured from different electronic fruits (drop height 10 cm in different fall directions onto steel or PVC).

Mikras implanted in a real potato tuber recorded an average peak acceleration of 110 g when laterally dropped from 10 cm onto a steel plate (Figure 6). The peak acceleration was almost doubled with Mikras implanted in a synthetic potato dummy. Similarly IRD devices and TuberLog each recorded higher peak acceleration values of nearly 200 g. This means that the synthetic casing material was in general more firm than the real tuber material. The difference in average peak accelerations between synthetic casing material and a real tuber might be less extreme when Mikras is implanted in turgescent freshly harvested tubers of higher firmness. In past free-fall experiments we observed an overall reduction of peak force values of 10% to 20% in potatoes which have been in storage for a longer period of time (over 8 months, data not published).

Repetitive test falls of electronic fruits produced consistent impact measurement values, which was shown by low standard deviation of their peak acceleration (up to 14 g).

Smart Spud with two different covers recorded much lower values than the other devices. Jaren *et al.* [17] observed that IRD detected impacts from very low drop height (2 cm), in contrast to Smart Spud which did not record those low impacts.

**Figure 7.**
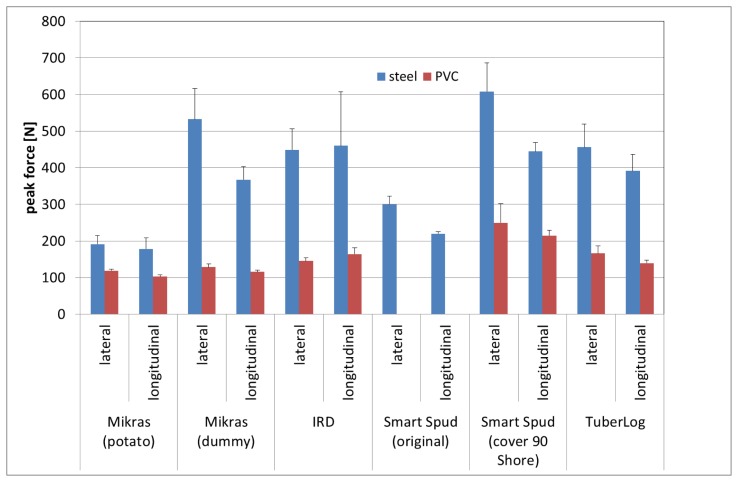
Peak force (drop simulator) for impacts of different electronic fruits (drop height 10 cm in different fall directions onto steel or PVC).

Our data showed higher peak acceleration values when fruits were dropped onto their flat side rather than the apical area (Figure 6). Our peak force measurements (Figure 7) showed that the drop direction also had similar effects on measured peak force values. Different drop directions produced different contact area sizes. Dropping fruits onto their flat side produced a bigger contact area between the fruit and the baffle plate resulting in less deformation of the fruit. At the same time there was less damping material between the surface of the fruit and the acceleration sensor in this drop orientation of the fruit.

**Figure 8.**
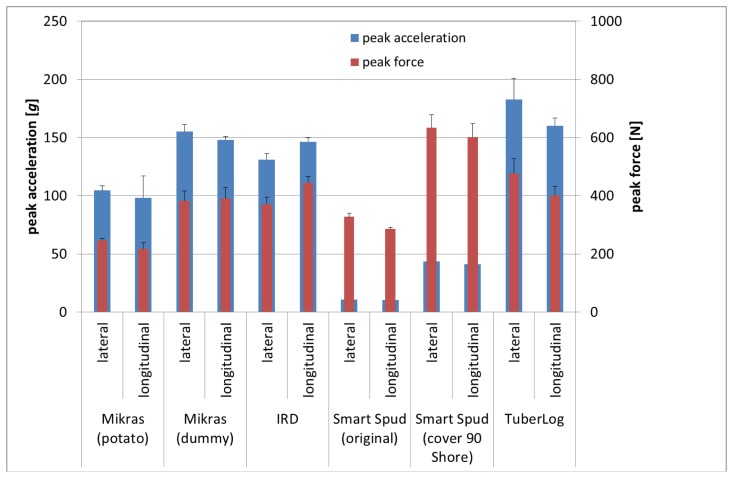
Peak acceleration (electronic fruit) and peak force (drop simulator) for drop heights of 25 cm in different fall directions onto PVC.

The influence of drop direction additionally depended on the damping properties of the baffle plate. When the electronic fruits hit on a weaker material such as PVC, comparable peak acceleration and peak force values were measured in both drop directions from 10 cm as well as 25 cm (Figures 6, 7 and 8).

When falling from 10 cm onto PVC foam the average peak acceleration was similar (50 g ± 20 g) for all electronic fruits, except Smart Spud (Figure 6). On the other hand differences between the electronic fruits were evident for falls onto PVC with drop height of 25 cm (Figure 8). Measurements with Mikras during the potato harvesting process resulted in similar peak acceleration values at critical points of the process when using a real potato tuber and the synthetic dummy [18]. Most likely the recorded impacts between 60 and 180 g occurred after dropping onto cushioned materials (e.g., other tubers) rather than on a firm material like steel.

The drop experiments show that the casing material of the devices and the cushioning material influences strongly peak acceleration and peak force values. The reason is that during the impact an important part of the impact energy is absorbed by soft impact material or soft shape of a produce and not converted into rebound energy [8].

The peak force values for falls from 10 cm drops onto steel were much lower for impacts of potato tubers than that of the other devices such as tuber dummy, IRD, TuberLog, and Smart Spud with cover of 90° Shore hardness. The lower values of Smart Spud (original) indicate that this cover material has very similar mechanical properties of real potato tubers (Figure 7). The high peak force values measured when the dummy with Mikras, IRD and Smart Spud (Grimme shape) were falling onto steel from 10 cm result beside firmness of the casing from the high mass of these devices.

The relationship of measured peak forces (drop simulator) and the peak force calculated from the impact acceleration measurements of the electronic fruits (Table 2) was very prominent (up to 10) for Smart Spud with both different covers. This means that impact acceleration measured with this device results in an underestimation of the actual mechanical load. The calculated relationships (*F_m_*/*F̂_c_*) of other electronic fruits were between 0.85 and 1.34. Values of the Mikras implanted in the dummy, IRD and TuberLog showed higher deviation from the value 1 for falls on steel than on PVC. This means that determination of impact force by impact acceleration measurements with electronic fruits is more accurate when hitting onto weak rather than firm material.

### 3.2. Detection of Impacts during Run through a Processing Line Simulator

During 10 runs in the processing line simulator the number of recorded single impacts was very different for the tested electronic fruits with threshold setting of 30 g. TuberLog registered the highest number of impacts of all electronic fruits where Smart Spud recorded the least impacts, namely less than half of TuberLog. Mikras implanted into a dummy recorded a very similar number of impacts to IRD.

The number of impact measurements was higher during fast conveyor velocitiy (0.35 m·s^−1^) compared to slow one of 0.19 m·s^−1^ for all tested devices except Mikras (Table 3). We suppose that this is caused by different levels of sensitivity or different recording modes of the acceleration sensors. In addition shapes and mass of the electronic fruits, as well mechanical properties of the cover material influence the number of recorded impacts.

For example, the egg-shaped Smart Spud rolls easier on a sloped conveyor belt compared to potato-shaped devices such as IRD, TuberLog, and Mikras implanted into a dummy or a real potato. The highest time duration during our tests was recorded by the egg-shaped Smart Spud, compared to real potatoes and potato-shaped devices such a dummy, IRD and TuberLog (Table 3).

**Figure 9.**
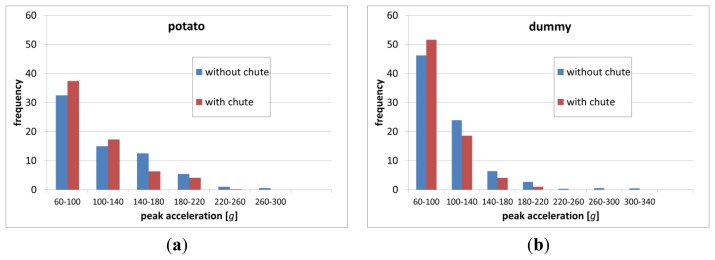
Frequency distribution of impacts with peak acceleration of ≥60 g measured with Mikras in a real potato (**a**) or in a dummy (**b**) during 10 runs through a processing line simulator (conveyor speed 0.35 m·s^−1^).

**Figure 10.**
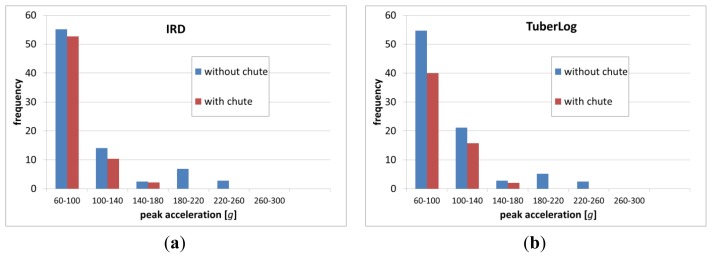
Frequency distribution of impacts with peak acceleration of ≥60 g measured with IRD (**a**) and TuberLog (**b**) during 10 runs through a processing line simulator (conveyor speed 0.35 m·s^−1^).

Occurrences of black spot disease of potato tubers after their mechanical load are of great economic importance. They depend on numerous factors such as the tubers' physical and physiological properties [19]. In our previous tests we observed black spot disease appear after peak acceleration impacts measured with Mikras in the range of at least 50 g to 100 g, depending on the size of the potatoes (data not published). Therefore our impact measurement histograms during processing line measurements (Figures 9 and 10) only show impacts with peak acceleration values greater or equal to 60 g.

Frequency distribution results show that Mikras implanted into a potato or a dummy, IRD, and Tuberlog have similar estimation of mechanical load when run through the processing line simulator. This is true for test with and without the effects of an impact reducing chute.

From all tested devices Mikras implanted into a real potato or a dummy have shown the widest range of peak acceleration values (up to 320 g) during runs though the processing line simulator at conveyor speed of 0.35 m·s^−1^ (Figure 9). During the same tests, TuberLog and IRD recorded lower peak acceleration values up to 240 g, however they have detected more frequent impacts of lower peak acceleration values of 60–100 g (Figure 10). Inserting a chute at the highest drop step of 34 cm mainly reduced the number of impacts with higher peak acceleration values (≥140 g).

At conveyor speeds of 0.35 m·s^−1^ Smart Spud with the original cover and with the cover of 90° Shore hardness recorded up to 25 impacts of peak acceleration between 20–60 g and only up to one impact of peak acceleration of 60–100 g. No impacts of higher peak acceleration values were recorded.

**Figure 11.**
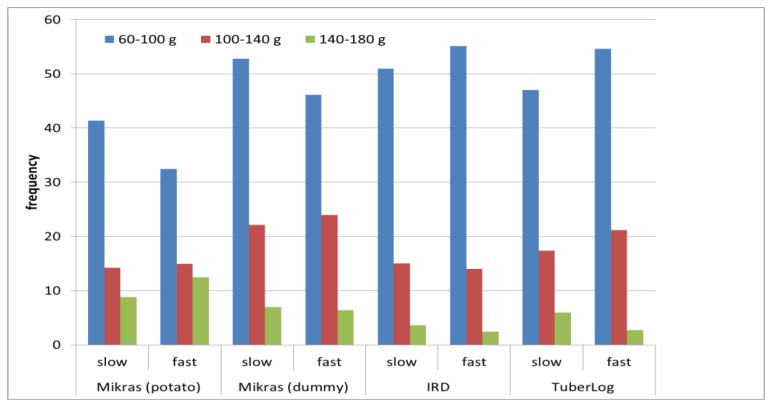
Frequency of impacts with peak acceleration between 60 and 180 g measured with different electronic fruits during 10 runs through the processing line simulator without chute with slow and fast conveyor speed (0.19 m·s^−1^ and 0.35 m·s^−1^).

Changing the conveyor velocity between 0.19 m·s^−1^ and 0.35 m·s^−1^ reduced or increased the frequency of impacts up to 20% in the different classes of peak acceleration for different electronic fruits (Figure 11).

**Figure 12.**
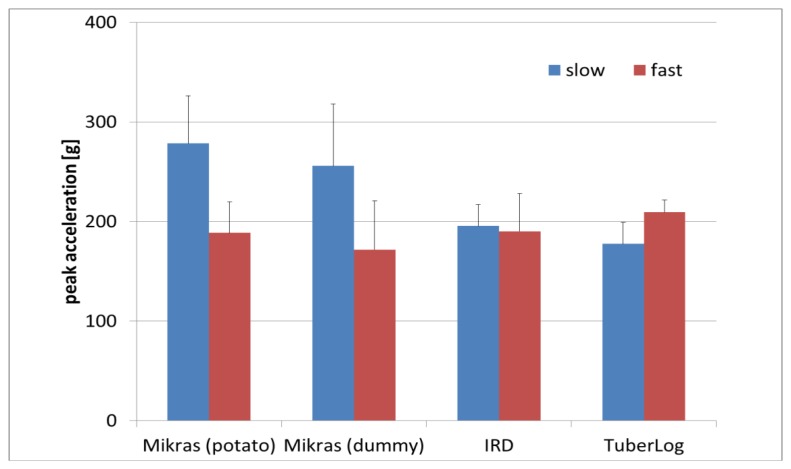
Mean values of peak acceleration at the highest falling step (34 cm) of the processing line simulator without chute with slow and fast conveyor speed (0.19 m·s^−1^ and 0.35 m·s^−1^).

At the maximum drop height of 34 cm, higher conveyor velocity led to lower peak values for drops of Mikras in real potatoes and dummies compared to IRD and TuberLog (Figure 12). The faster movement of the conveyor may cause an attenuation of this impact. This effect was not observed for IRD, which is likely caused by their physical features such as their heavier weight, shape, and form. No explanation could be found why TuberLog did not register lower peak acceleration values with fast conveyor velocity, notwithstanding it has a similar shape and weight as a potato dummy.

## 4. Summary and Conclusions

Peak acceleration values measured by the Mikras unit in real potatoes dropped from a height of 10 cm onto a steel plate were much lower than those measured with Mikras in a dummy, IRD, and TuberLog. This effect, which is due to the higher firmness of synthetic shapes compared to real potatoes, does not occur when a weaker impact material (PVC) is used. Peak acceleration values measured with SmartSpud during free-fall experiments were notable lower than values of other electronic devices. The reason for this is not clear. When real tubers or electronic fruits are dropped onto steel on their flat side impacts lead to higher peak acceleration values than when dropped onto the apical region. This is most likely caused by the higher material thickness and smaller contact area in the longitudinal drop orientations.

Tested electronic fruits with synthetic shapes have shown overall higher peak force values when dropped onto steel compared to those of real potatoes due to their higher firmness and mass. The cover of SmartSpud devices seems to exhibit very similar elastic compliance of real tuber shown by their similar measured peak force values.

The determination of the impact acceleration during runs in the processing line simulator produced comparable results for Mikras implanted into real potatoes or a dummy, IRD and TuberLog. SmartSpud on the other hand recorded much lower number of impacts and smaller impact acceleration values.

A prediction of produce bruising probability based on measurements with these devices requires specific determination of relationships between impact acceleration and produce damage. Mikras implanted into real potatoes or dummies, IRD and TuberLog are very suitable for determining critical points of mechanical load during harvesting and processing.

The compared devices differ in their possibilities of data evaluation and handling during the measuring operations. Regarding the practical application of the devices handling of TuberLog system is easy for the user. For research purposes it was Mikras and IRD that provided all necessary impact acceleration values (in three axes) related to the time throughout the entire measuring process. For practical evaluation of impacts during the harvest and postharvest processes, peak acceleration values exceeding a certain threshold may be sufficient.

## Figures and Tables

**Table 1.** Different measuring devices with selected specific settings for the application in this study.

**Parameter**	**Mikras**	**IRD**	**Smart Spud**	**TuberLog**
Shape	Real product in which Mikras is implanted	Flattened sphere	Egg-shaped	According to longish-shaped potato tuber
Material of skin	Real product tissue and skin plastic (dummy)	Plastic	Plastic	Plastic
Mass	14 g + mass of real product: potato tuber 203 g (free-fall test), 220 g (processing line simulator test) dummy 213 g	270 g	Depending on shape 274 g (original) 314 g (Grimme shape)	200 g
Dimension	42 mm × 13 mm × 13 mm (implant) 81 mm length, 59 mm diameter (potato) 97 mm length, 66 mm/ 53 mm diameter (dummy)	90 mm length, 79 mm/52 mm diameter	108 mm length, 73 mm diameter (original shape) 108 mm length, 75 mm diameter (Grimme shape )	90 mm length, 65 mm/50 mm diameter
Hardness	90° Shore A (dummy)	Not specified	Not specified (original shape) 90° shore a (Grimme shape)	80°–85° shore a
Measuring parameter	Triaxial gravitational acceleration	Triaxial gravitational Acceleration	Triaxial gravitational Acceleration	Triaxial gravitational Acceleration
Scanning rate	3 kHz	4 kHz	Not specified	3 kHz
Threshold for triggering measurement	30 g *	30 g *	Not specified	30 g *
End of measuring range	200 g *	500 g *	Not specified	250 g *
Resolution/Accuracy (manufacturer)	∼1 g */Not specified	∼ 2 g */3%	Not specified	0,1 g */±1 g *

* multiple of g (gravitation).

**Table 2.** Relation of measured peak force with drop simulator (*F_m_*) to calculated peak force (*F̂_c_*) for the falls from 10 cm height onto steel or PVC foam.

**Electronic fruit**	**Drop direction**	**Relation *F****_m_*/*F̂_c_*	
		steel	PVC foam
Mikras (potato)	lateral	0.85	1.03
	longitudinal	1.07	1.08
Mikras (dummy)	lateral	1.27	0.97
	longitudinal	1.22	1.05
IRD	lateral	1.03	1.01
	longitudinal	1.36	1.11
Smart Spud (original)	lateral	9.88	no data
	longitudinal	10.86	no data
Smart Spud (cover 90 Shore)	lateral	4.65	11.21
	longitudinal	5.64	17.55
TuberLog	lateral	1.00	1.15
	longitudinal	1.54	1.09

**Table 3.** Number of impacts and time duration detected during 10 runs through the processing line simulator without chute (threshold value for peak acceleration 30 g, conveyor speed slow = 0.19 m·s^−1^ and fast = 0.35 m·s^−1^).

**Conveyor Velocity**	**Mikras (Potato)**	**Mikras (Dummy)**	**IRD**	**Smart Spud (Original)**	**Smart Spud (Cover 90 Shore)**	**TuberLog**

Number of impacts						

slow	117	136	129	50	69	163
fast	102	117	139	76	78	170

Time (s)						

slow	172	181	172	243	218	167
fast	98	86	105	164	126	102
